# Speciation, pattern recognition and the maximization of pollination: general questions and answers given by the reproductive biology of the orchid genus *Ophrys*

**DOI:** 10.1007/s00359-019-01350-4

**Published:** 2019-05-28

**Authors:** Hannes F. Paulus

**Affiliations:** Department of Integrative Zoology, Faculty of Life Sciences, University of Vienna, Althanstr.14, 1090 Vienna, Austria

**Keywords:** Selection, Reproductive success, *Ophrys*, Learning behavior

## Abstract

Pollination syndromes evolved under the reciprocal selection of pollinators and plants (coevolution). Here, the two main methods are reviewed which are applied to prove such selection. (i) The *indirect* method is a cross-lineage approach using phylogenetical trees to understand the phylogeny. Thus, features of single origin can be distinguished from those with multiple origins. Nearly all pollination modes originate in multiple evolutionary ways. (ii) The most frequent pollinators cause the strongest selection because they are responsible for the plant’s most successful reproduction. The European sexually deceptive orchid genus *Ophrys* provides an example of a more *direct* way to prove selection because the attraction of a pollinator is species specific. Most members of the genus have remarkably variable flowers. The variability of the signals given off by the flowers enables the deceived pollinator males to learn individual flower patterns. They thus avoid already visited *Ophrys* flowers, interpreting them as females rejecting them. As the males will not return to these individually recognizable flowers, the pollinators´ learning behavior causes cross-pollination and prevents the orchid’s self-pollination.

## Introduction

Most advances in pollination biology have resulted from interdisciplinary research combining ecological and evolutionary perspectives. Several approaches have been essential for understanding the functional ecology of floral traits, the dynamics of pollen transport, competition for pollinator services, and patterns of specialization and generalization in plant–pollinator interactions. Understanding plant–pollinator interactions requires and invites a variety of viewpoints and conceptual approaches, ranging from descriptions of animal behavior and their contributions to pollination success to floral evolution. Many of the results of evolutionary approaches in pollination biology are summarized in some very informative textbooks (Harder and Barrett 2006; Willmer 2011) or the many special articles in Patiny (2011); for a more special consideration have a look in Chittka and Thomson (2001) or Waser and Ollerton (2006).

These approaches reflect the two historic starting points for the discipline. One approach emphasized detailed observation of floral mechanisms and the natural history of the ecological relationships between plants and pollinators. It originated with pioneering work by Sprengel (1793) and Müller (1873). An interesting second approach focuses on evolutionary processes that might affect and be affected by pollination. Pollinators act as selection factors attracted by signals emitted by the flower to increase its reproductive success above that of other individuals of the same species. Some plants operate with signals similar to sexual traits in a pollinator´s courtship behavior to attract it and to thereby guarantee fertilization. The specific patterns of pollinator visitation influence important aspects of the pollinator´s mating system and minimize self-pollination of the plant. A special case in this respect is the sexual deception by some orchid genera like that by the European *Ophrys* species (Pouyanne 1917; Kullenberg 1961, 1973; Paulus 2006, 2007; Schiestl 2011).

## General questions

Research in pollination biology has been at the forefront of theory testing and model building. In evolutionary biology, pollination systems have provided some of the best tests of theories of evolution by natural selection and the adaptive nature of floral traits (Levin 1985; Campbell 1985; Campbell et al. 1996; Schemske and Bradshaw 1999). One of the main questions in this respect is whether pollinators act as selective partners triggering most of the flower evolution. There are two different ways to find answers: (i) one mainly comes from reconstructions of phylogeny and evolutionary processes (e.g. DeWitt Smith 2010), (ii) the other relies on measurements of reproductive success as a function of pollinator behavior (e.g. Paulus 1988, Gervasi and Schiestl 2017). The origin of pollination modes in specific plants can possibly be explained using the results of phylogenetical trees. These can help to understand how often and in which ways a particular pollination syndrome evolved. A quantitative measure of selective pressures mainly needs long-term observations in the field or even better experimental work.

### Flowers and their specific pollination modes

Most of the angiosperm flowers require help in transporting their pollen to the stigma of another flower of the same species. They borrow it from different animals (zoogamy: pollination by animals; entomogamy: pollination by insects). Exceptions are species pollinated by wind (anemogamy) (most gymnosperms, secondarily some groups of angiosperms) or obligate selfers (autogamy). In Europe, most pollinators are insects. Many of them are very selective regarding the flowers visited.

Whereas most angiosperms have hermaphroditic flowers, gymnosperms primarily have separate male and female flowers. They use the wind for pollination. To ensure fertilization, male flowers have to produce enormous amounts of pollen grains. As pollen contains a lot of nitrogen, a strongly limited resource, its production is a costly process. Pollination by insects evolved during the early Mesozoic era, possibly first by some of the pollen-eating beetles which had been well established since the Perm period. As some beetles fed on the seeds of gymnosperms, some of these developed sheets protecting their seeds, which became a crucial characteristic of the later angiosperms. Pollen feeding alone was not enough to ensure pollination as these beetles visited only the male flowers. Female flowers were not attractive, as nectar evolved much later. The solution was to evolve bisexual flowers, the next special characteristic of most angiosperms.

### Evolution of flower signals

The rapid expansion of insect pollination was due to the emergence of “clever” attractants, such as flower shape connected with colored displays and many different scents. The flower colors were selected by the pollinators, co-evolving with their visual system (Schiestl and Johnson 2013). There must have been strong competition between the early flowers for insects and later also other pollinators. As a consequence, the signals evolved in many different directions. The motivation of the flower visitors had to be rewarded with food. In most cases, this is protein-rich pollen and nectar, providing simple sugars such as fructose, saccharose and glucose. These are ideal energy suppliers which can be directly transferred into muscle activity during flight. Bees (Apoidea) evolved shortly after the flowering plants and diversified into higher lineages contemporaneously with the radiations evolving within the angiosperms. Major bee lineages (i.e., families) were presumably established by the late Cretaceous (Cardinal and Danforth 2013; Cappellari et al. 2013; Plant and Paulus 2016; Sann et al. 2018). Bees visit flowers not only to feed themselves. Instead, they are specialised in collecting and storing pollen and nectar, as food for their larvae. This significantly increased the frequency of flower visits. Bees evolved to be very efficient pollinators and most of the angiosperm species today are pollinated by one or several of the many hundreds of different bee species. As a consequence of the competition for pollinators, the rich diversity of today’s flower types has evolved which also triggered multiple evolutions of new pollinator animals. This process of reciprocal selection is accompanied by many reciprocal adaptations (Gilbert and Raven 1975; Paulus 1978; Janzen 1980). The result is a remarkable diversity in butterflies, bees, flower visiting birds or bats, which would have never evolved without their flowers. Because of the development of more and more specialised pollination methods, signals had to become more differentiated. As bees, like most insects, are able to see colors, even UV, flowers developed many different color patterns and indeed UV patterns which we as human beings are unable to see (Dyer et al. 2012). Many of these UV patterns together with yellow markings are interpreted as imitation of the stamina or the two-lobed anther because they mainly occur on flowers with hidden androecium. Therefore, these spots are replacement signals instead of the normally visible stamens (Osche 1979, 1983; Paulus 1988; Lunau 1990, 2000; Lunau et al. 2017). Importantly, when interpreting the color of a particular flower we always have to ask for the visual system of the pollinator. The same applies to all the other sensory modalities. Olfactory flower signals in most cases help the pollinator to find flowers at larger distances. Another field of research comes from findings that pollen transfer is also influenced by electrostatic forces because charged pollen settles more effectively on stigmas than uncharged pollen (Hardin 1976; Buchmann and Hurley 1978; Vaknin et al. 2000). The study of pollinator attraction by physical forces is a field that depends on much more interdisciplinary working and interdisciplinary funding models (Moyroud and Glover 2017).

### Evolution of pollination syndromes

Pollination syndromes are a sum of special flower characteristics evolved by the selection of a special and more or less closely related group of pollinators and depending on how strong their influence on the reproductive success of the flower is. A critical review of this old theory is summarized in Ollerton et al. (2009). A detailed understanding of the most relevant flower characteristics tells us who the main pollinators will be and to distinguish bat, bird, moth, butterfly or bee pollinated flowers from each other. The respective syndromes are chiropterophily, ornithophily, phalaenophily, psychophily and melittophily. Flowers pollinated by bees are mostly yellow or blue-violet, flowers visited by butterflies are mainly bluish-red or sometimes pure red, those visited by moths are white, whereas bird flowers are mostly pure red. In general, these colors are adaptations reducing competition. Thus, the red flowers visited by birds appear black to bees, to give an example. It is interesting to realize that on the Hawaii islands without or with only a few native wild bees species bird flowers are more or less blue (Cronk and Ojeda 2008; Pender et al. 2014). Night blooming bat flowers in South America have more or less reduced colors, such as different shades of green, because these bats (Phyllostomidae) locate flowers via their smell and ultrasonic echolocating signals (von Helversen and von Helversen 1999; Datzmann et al. 2010; Simon et al. 2011). In Europe and North America bee flowers dominate. The species-rich members of Apoidea are very effective pollinators because they visit flowers not only for themselves but mainly to collect pollen and nectar for their brood. Many genera are more or less seasonally staggered over the year to reduce competition, first summarized by Baker and Hurd (1968) and in more detailed scenarios in Mitchell et al. (2009). Among these in particular, the eusocial ones are even more effective because they produce much more larvae and they collect additional nectar to store for bad times. This had and still has a strong influence on the diversity of flower types. As color vision in insects and especially in bees existed before the evolution of most angiosperms, insects triggered colored flowers in multiple evolutionary ways (Chittka and Menzel 1992; Chittka et al. 2001; Dyer et al. 2012). The same is true in bird-pollinated flowers where pure red is common (Rodríguez-Gironés and Santamaría 2004; Burd et al. 2014). In the temperate latitudes of Eurasia, especially in cooler mountain areas, bumblebee species are among the most important pollinators. As generalists, they also are a good example regarding the reduction or avoidance of competition by spreading on a diversity of flower types enabled by differences in tongue lengths and learning behavior (Pyke 1978; Inouye 1978; Heinrich 1976, 1979; Neumayer and Paulus 1999; Essenberg et al. 2015).

According to an early knowledge, nearly all pollination syndromes are the result of multiple evolutionary convergences (Vogel 1954). It seems to be a general principle for closely related plant species to specialise on different pollinators during a radiation process. This is the best way to minimize competition and to build different ecological niches by character displacement (Levin 1985). A good example is the old comprehensive investigation of all the pollination methods within the plant family Polemoniaceae (*Phlox* family) by Grant and Grant (1965). They found out that the original pollination method was bee pollination. From this kind of pollination, many different and new pollination mechanisms evolved. After a comprehensive phylogenetical analysis of most of the genera and many different species, it could be extrapolated that ornithogamy independently evolved at least 7 times, butterfly pollination at least 3 times, from this level several times moth pollination, finally 11 times pollination by flies with a long proboscis (Bombyliidae, Sepsidae, etc.), twice beetle pollination and even once bat pollination in the genus *Cobaea*. Local species communities within the Polemoniaceae consist predominantly of different pollination types (Plitmann and Levin 1990). A comparable investigation was recently published for the plant family Apocynaceae (Ollerton et al. 2019).

Apart from avoiding competition between pollinators, evolutionary constraints play an important role in channelling selection processes. Tropical rainforests are characterized by an enormous biodiversity, including the number of different tree species per square meter. If the next specimen of a special tree species grows far away then pollination can only be done by animals able to fly such long distances. Those so-called long-distance pollinators are birds (hummingbirds, sun birds, etc.), large bees (Anthophorinae) and during the night, bats (Phyllostomidae: Glossophaginae in America, flying foxes: Megaloglossinae in Africa and South Asia). By plotting the occurrence of bat pollination in the phylogenetical tree of the Angiospermae, it can be seen that even within the major family groups this method of pollination has evolved independently at least 27 times (Fleming et al. 2009). Besides this, alone within the different pantropical plant families of Malvales, here within the subfamily Bombacaceae, the tree genera *Adansonia, Kigelia* (Africa), *Bombax* (pantropical), *Ceiba* (pantropical), *Ochroma* (S. America) or *Pseudobombax* (S. and Central America), the method of bat and bird pollination has evolved multiple times. Within the same plant species *Pseudobombax ellipticum*, one finds red flowers for hummingbirds and white flowers for phyllostomids, depending on their distribution. Remarkably, different bat pollinators show the same sensitivity for the odorants emanating from bat flowers (von Helversen et al. 2000; Knudsen and Tollsten 2008).

### Evolution and pollinator selection

Although the diversity of pollination syndromes can only be a consequence of selection processes caused by flower-visiting animals, it is difficult to demonstrate this directly by observations or experimental work. A new experimental work in this respect was published by Gervasi and Schiestl (2017) for *Brassica rapa* with fast cycling plants to demonstrate adaptive evolution driven by different pollinators. The study shows pollinator-driven divergent selection as well as divergent evolution in plant traits. Plants pollinated by bumblebees evolved taller size and more fragrant flowers with increased ultraviolet reflection. Bumblebees preferred bumblebee-pollinated plants over hoverfly-pollinated plants at the end of the experiment, showing that plants had adapted to the bumblebees’ preferences. Plants with hoverfly pollination became shorter, had reduced emission of some floral volatiles, but increased fitness through augmented autonomous self-pollination. But usually, we only can reconstruct past evolutionary processes by comparing different patterns of characteristics, interpreting them as supposed evolutionary lines. This corresponds to the method of finding homology, especially by the criterion of transition series. But the assumption that these transition series are a consequence of different selective processes is not more than a plausibility or probability. But how we can get a more direct evidence?

Selection can be defined as the difference of reproductive success between two individuals of the same species caused by differences in geno- and phenotype. A valid approach to solve the problem of proving selection is to find out that and why different individuals within a population have different reproductive success.

## Evidence for selection

### Indirect evidences

Especially high numbers of convergences seen in a particular pollination syndrome speak for many selective processes. Appealing are those cases where alternative explanations are very improbable. An example is the species composition within the plant genus *Thalictrum* (Ranunculaceae). European species of this genus are characterized by lacking the whole perianth because they are pollinated by wind. But there are some species which are entomogamous and have colored stamina instead of a colored perianth (which is missing). Two species are common in Europe, one with yellow stamina (*Thalictrum flavum*) and another with purple stamina (*Thalictrum aquilegifolium*). Why the perianth is lacking in spite of pollination by insects? Petals in zoogamous flowers are normally colored. As many species of the genus *Thalictrum* have normal colored petals and greenish stamina, the best explanation is that the species without petals and colored stamina evolved from wind-pollinated precursors. Support for this hypothesis comes from the phylogenetical tree of the genus by Soza et al. (2012). All species with colored stamina and lacking crown leaves have wind-pollinated species as sister groups. This means that the original pollination syndrome of *Thalictrum* within the Ranunculaceae is insect pollination, but several times and independently secondary wind pollination evolved. From these precursors again new entomogamy evolved several times. As these precursors had reduced their perianth in adaptation to wind pollination, selection for a new insect pollination colored those structures in the flower which had not been reduced before. A similar example exists in the genus *Plantago* (Plantaginaceae) with primarily insect-pollinated species and a secondary transition to wind pollination. *Plantago media* is tertiary insect pollinated in the same manner as shown for *Thalictrum*. *Plantago media* completely lacks the perianth and instead, it has also colored stamina.

### Direct evidence

In case of direct evidence for selection, one has to demonstrate that within a population of a given species different genotypes exist with different pollination success depending on different pollinators. A good example is *Mimulus aurantiacus* (Phrymaceae) in the USA. Along coastal regions, its flowers are mostly red, whereas towards the mountains they are yellow. In the areas in between one finds mixed color forms (Streisfeld and Kohn 2007). The explanation is that in the coastal regions hummingbirds are the most frequent pollinators selecting plants with red flowers, whereas in the mountain regions large bees are the most important pollinators. Similar cases of color polymorphism were found in southeastern Mediterranean regions with *Ranunculus asiaticus* or *Anemone pavonina*. Both species show a distinct color polymorphism with flowers ranging from blue-violet, yellow and white to pure red. In regions where members of the beetle genus *Pygopleurus* (= *Amphicoma* pars) (Scarabaeoidea, Glaphyridae) are pollen eaters and pollinators, selection is for red flowers of *Ranunculus asiaticus* and *Anemone pavonina* implying that the beetles see in the red (Sommer 2010; Martínez-Harms et al. 2012). Where these beetles are rare or lacking, bees are the most common pollinators and select for other colors (Dafni et al. 1990, Keasar et al. 2010, Sommer 2010, Streinzer et al. 2019). The frequencies of color forms in different populations follow the frequency-dependent selection principle first formulated by Stebbins (1970) for pollination systems. The most frequent successful pollinators perform the strongest selection. This was shown even at a community level as dominant pollinators drive even non-random community assembly (Kemp et al. 2018). Hummingbirds waiting near bat flowers for them to start nectar production in the early evening were observed many times. Some hummingbirds pollinate the bat flower. But their quantitative contribution to the reproductive success is small as long as most visits come from the later flying bats. However, there might be situations where bats become rare and hummingbirds more common. In these cases, the birds will attain a leading role in selection. Plant individuals starting their nectar production a little bit earlier will have more reproductive success. Then, a kind of directional selection starts and the hummingbirds will transform the former bat flower more and more into a bird flower. The former bat flower will die out. There are many examples which seem to support this view. In many genera, there are flowers pollinated by bats as well as by hummingbirds. A phylogenetic tree of those genera demonstrates that a change from bat pollination to bird pollination and vice versa has occurred several times (von Helversen 1993; Fleming et al. 2009). An example is the tree *Erythrina fusca* in North Columbia, a member of the pantropical genus *Erythrina* (Fabaceae) with species nearly all pollinated by birds. Their flowers are light reddish brown, produce nectar at night and are visited by Glossophagine bats (Paulus 1978).

Despite recent progress in the ecology of pollination, the sensory floral traits that are important for communication with pollinators (for example, color and scent) have not been assessed in an unbiased, integrative sense within a community context. New results reveal a coordinated phenotypic integration consistent with the sensory abilities and perceptual biases of bees, suggesting potential facilitative effects for pollination and highlighting the fundamental importance of bees in Mediterranean-type ecosystems (Kantsa et al. 2017). Despite progress in understanding pollination network structure, the functional roles of floral sensory stimuli (visual, olfactory) have rarely been addressed comprehensively in a community context, even though such traits are known to mediate plant–pollinator interactions (Sargent and Otto 2006). There is an interesting new approach using a large data set of many functional flower traits to understand more of pollinator—flower interactions at a community level (Kantsa et al. 2018). In many cases of plant communities, it is difficult to separate between competition of single members within a community and the different kinds of facilitation (mutualisms or commensalisms). The following section presents examples of obvious competition between different species of the same genus of orchids and even within the same species.

## Observations and experimental work with orchids of the genus *Ophrys*

**Fig. 1:**
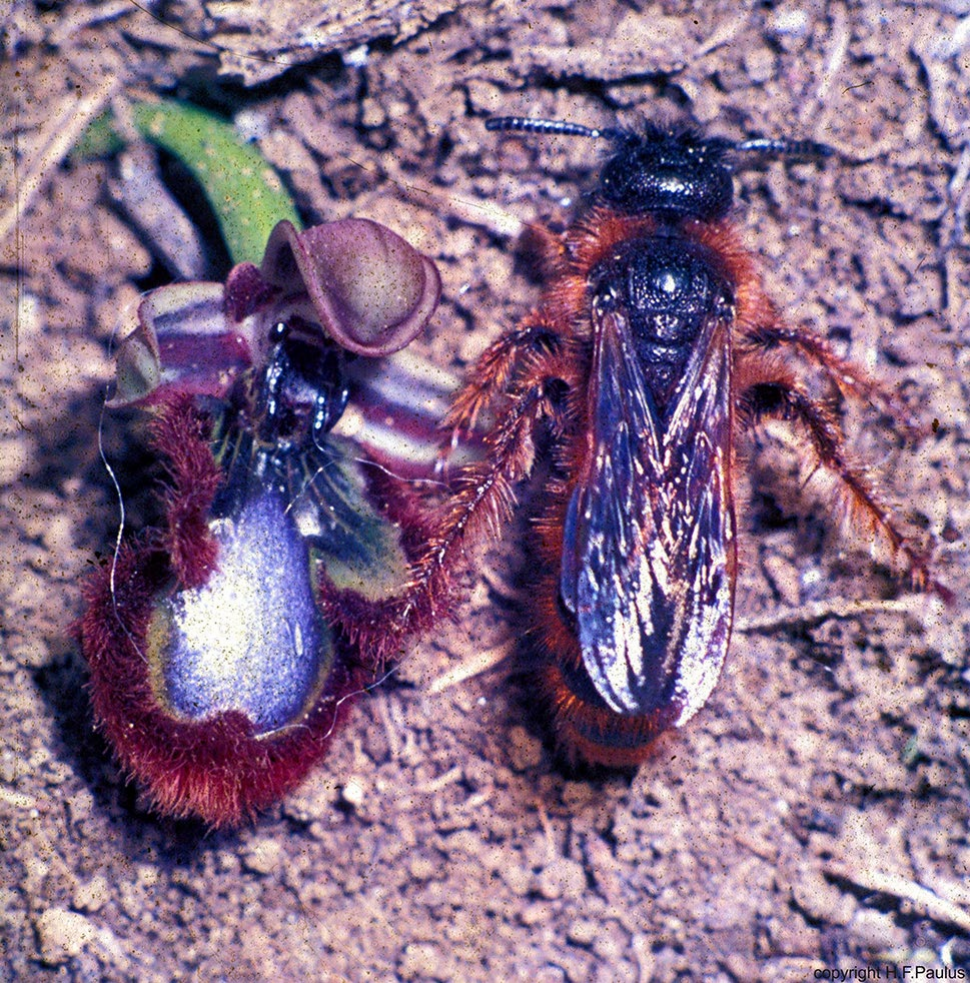
Flower of *Ophrys speculum* beside the female of the pollinator wasp *Dasyscolia ciliata* (Hymenoptera, Scoliidae). Note the strong visual similarity. The blue shining pattern of the flower imitates the blue shining wings of the female wasp. The reddish hairs along the flower lip are the imitation of the respective body hairs of the female (from Paulus 1978)

To find a better connection for the interpretation of past selections on flower syndromes, it might be important to demonstrate how directly selection can work. Because of the high specialization in pollination biology, most orchids could be a good example to investigate reproductive success in connection with intraspecific variability. Most species of *Ophrys* achieve pollination by means of sexual deception, and the behavior displayed by the male insects pollinating them is called pseudocopulation. The flowers imitate the signals which release mating behavior in the pollinator species, serving species recognition and the prevention of hybridization (Pouyanne 1917; Kullenberg 1961, 1973; Paulus 1978; Paulus and Gack 1981, 1990; Paulus 2006). In addition, the signals may play an important role in mate selection. Three basic sets of signals or stimuli are critical here—olfactory, visual (Fig. 1) and tactile.

Each set of signals represents a more or less complex pattern having the attributes of key stimuli and activating innate releasing mechanisms. It will be shown in the following that *Ophrys* flowers are able to imitate all of these signals and thereby successfully attract particular species of pollinator males, deceive them and profit from the transfer of their pollinaria.

### Olfactory signals (Fig. 2)

Female insects frequently produce chemical attractants (sexual pheromones) to attract a sexual partner. The males of each species have evolved their own innate releasing mechanisms which enable them to pick exactly that blend which their females emit from thousands of possible odor-stimuli. The sexual pheromones produced by the labellum of the orchids act as long-range attractants, guiding males into the proximity of flowers. At close range, however, they trigger a sexual response. Male pollinator insects try to copulate with the female-like labellum (pseudocopulation). In glandular cells under the labellar surface of each *Ophrys* flower, hundreds of odor components are produced which according to their mixture and concentration make up the species-specific odorant bouquet (perfume). The chemical substances include, above all, terpenoid, long-chained aliphatic hydrocarbons, aldehydes, ketones and 1-, 2-alcohols as well as cyclic (aromatic) compounds. All of these substances are commonly used as scents and fragrances in the plant and animal kingdoms. Since 1978, Bergström (1978), Borg-Karlson (1990) and others have documented the chemistry of the olfactory attraction based on analyses of flower and bee odorants. However, for a long time it was difficult to make any sense out of the astoundingly long list of volatile odorant compounds present in *Ophrys* flowers (more than 100 different volatiles), especially since no agreement was found in qualitative studies of the odorant chemistry between the flower and the pollinator (Borg-Karlson 1990). Behavioral experiments utilizing synthetic copies of compounds produced by *Ophrys* flowers have shown that only certain volatiles are active in stimulating mating behavior in males.

**Fig. 2:**
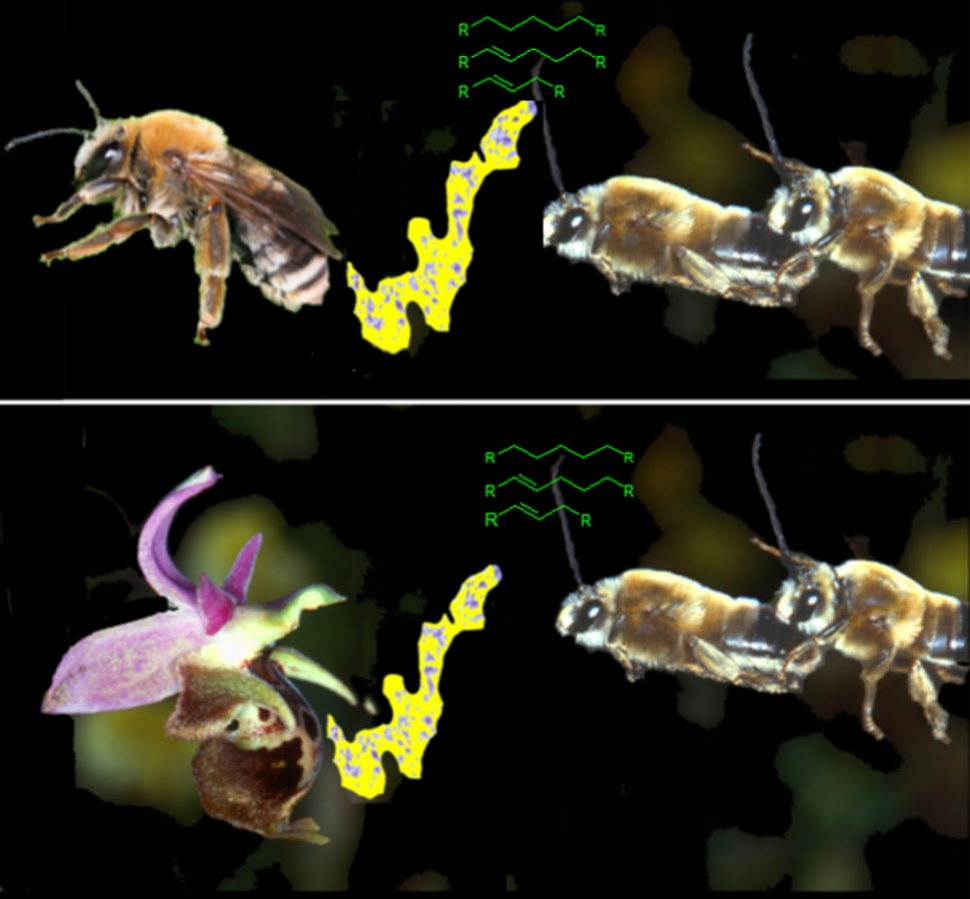
Species of the orchid genus *Ophrys* in Europe mimic females of solitary bees willing to mate. These female bees produce species-specific sexual pheromones to attract their conspecific males. An *Ophrys* flower produces identical sexual pheromone compounds to attract the male bees for pollination. Here *Ophrys heldreichii* (Crete) and its pollinator males *Eucera berlandi* are shown as an example (modified from Paulus 2018)

Based on numerous field observations, it becomes obvious that innate behavior mechanisms of male insects are released by “female” scents copied by *Ophrys* flowers. The *Ophrys* flowers will excite males even long after the females have emerged (Paulus 1988, 2007; Paulus and Gack 1990).

#### The odorant bouquet, a perfectly copied signal

Since previous attempts had failed to identify which of the many hundreds of volatile odorants cause the male to react a new procedure was called for. First of all, it was necessary to determine the chemical composition of the sexually attractive scent of the female insect to know what to search for.

Using a combination of gas chromatography plus electroantennographic detection (GC–EAD), gas chromatography plus mass spectrometry (GC–MS), as well as behavioral field tests, we could identify compounds that mediate male behavior in the orchid *Ophrys sphegodes* and its pollinator bee *Andrena nigroaenea* (Schiestl et al. 1999, 2000) as well as in *Ophrys speculum* and its pollinator wasp *Dasyscolia* (*Campsoscolia) ciliata* (Ayasse et al. 2003). We also tested the hypothesis that *Ophrys* flowers produce only “second-class” attractant compounds (Borg-Karlson 1990) that are less attractive than those of the genuine female insects.

This could be shown to be false by applying a combination of a gas chromatographic analysis of the odorants along with recordings of the electrophysiological response of the male antenna. The fragrance of both the orchid *Ophrys sphegodes* and its pollinator *Andrena nigroaenea* females was examined. Each type of odorant volatile was tested for an electrophysiological reaction on male antennae. The receptors on the antennae of the males responded to 16 volatile odorants found in both the flower and the female scents. In subsequent behavioral tests, a bouquet of exactly these 16 volatile compounds was presented to males flying in the field. They reacted to this mixture with specific copulation movements (Schiestl et al. 1999, 2000). The attractants of the flower and those of the female were identical. The *Ophrys* system of mimicry operates so well because the species-specific stimuli of the female scent are precisely identical to the flower’s scent. Apparently, many of the closely related *Ophrys* species utilize species-specific blends of the same hydrocarbons. The same principle of species-specific mate attraction also applies to various species of moths (e.g. Linn and Roeloffs 1995).

We studied closely related species complexes (*Ophrys fusca* s. lat. and *O. sphegodes* s. lat.). Both groups have many allopatric species with geographically disjunctive distribution but with more or less identical olfactory compounds (Stökl et al. 2005, 2008, 2009). Remarkably, in both species-groups the geographical forms can be interpreted as separate species. This conclusion is also based on diverging flower morphology and strongly supported by morphometric studies (Paulus 2001; Lowe 2011). The pollinator bee in all cases belongs to the same species. We hypothesize that these morpho-species have each independently acquired the same pollinator (Paulus 1998, 2001, 2006), and thus their odorant bouquets must be the same. The pollinating bee species are *Andrena nigroaenea* and *Andrena flavipes*. According to a comparative investigation, all of these *Ophrys* species attract bees with the same odorant bouquet (Stökl et al. 2005, 2008, 2009) which must have evolved several times independently.

### Visual signals

Once a male is near a female, he is able to visually perceive her optical signals which often are species specific. Some *Ophrys* flowers also imitate the shape and coloration of the pollinating bee´s female. A striking example even for the human eye is the mirror orchis (*Ophrys speculum*) and the female of the scoliid wasp *Dasyscolia ciliata*. This orchid species imitates the dark blue shining wings together with their UV reflection, the reddish brown body hairs and even the middle and hind legs (Fig. 1). If the same bee species pollinates in different parts of the Mediterranean area different, not closely related *Ophrys* species, then it is to be expected that these *Ophrys* species not only have similar odorant bouquets but also similar optical cues. Examples for such convergences are the Italian *Ophrys bertolonii*, the Greek *O. ferrum*-*equinum* and the Spanish *O. atlantica*. All these *Ophrys* species are pollinated by the same species of mason bee, *Chalicodoma parietina* (*Megachilidae*). Further examples are the Italian *Ophrys apulica*—the Greek *O. heldreichii*, both pollinated by the long-horned bee *Eucera berlandi,* or the Cypriot *Ophrys kotschyi* and the Cretian *O. cretica*, both pollinated by the bee *Melecta tuberculata* (Paulus and Gack 1986; Paulus 1988, 2007). Another example for the independent selection by the same wasp species as pollinator in three different *Ophrys* species is given by Paulus and Hirth (2011). Here, the crabronid wasp *Argogorytes fargei* or *A. mystaceus* pollinate the *Ophrys insectifera*, *O. cilicica* and *O. regis*-*ferdinandii* which are unrelated within the genus *Ophrys*. The results are very similar flower shapes untypical for the other members of the genus. Like in similar cases we had investigated (e.g. in the *Ophrys fusca* group: Stökl et al. 2005), it is to expected that all three species are working with identical sexual pheromones. The pinkish perigon of many *Ophrys* (see Fig. 5) species may increase visual contrast between the labellum (as the pretended female) and the background. Both green leaves and the dark brownish labellum provide only low or no chromatic information for bees, since colors like green and dark brown reflect more evenly among the entire visible light spectrum. In contrast, a bright pink has two reflection maxima in the blue and the orange to red part of the spectrum, respectively, and absorbs in the green part. Thus the dark labellum of an *Ophrys* appears visually conspicuous against the bright pink perigon for the pollinator males due to a strong chromatic and brightness contrast (Spaethe et al. 2007, 2010; Streinzer et al. 2010).

An interesting question is whether virgin females exhibit the same attractiveness as the *Ophrys speculum* flowers. For experimental tests, we had to obtain females of *Dasyscolia* (*Campsoscolia) ciliata* which were not yet mated and to offer them in a choice-test flowers of *Ophrys speculum* in addition to the males. These experiments showed that no essential differences in attraction existed. Even the female scent is not more effective than the flower’s (Paulus 2006, 2007).

### Tactile signals

Within *Ophrys*, we can separate two groups of pollination types: in one the pollinaria are removed with the head (group *Euophrys*); in the other, pollinaria are removed with the tip of the abdomen (group *Pseudophrys*). After the male has landed on the female, the tactile stimuli come into action and help the male to determine which end of the female body is the front and which is the back. For this purpose, the male perceives the direction of the female’s body hairs (Kullenberg 1961). The males must perform their orientation task quickly because of competing males and whoever is the fastest wins (scramble competition). To what extent the tactile stimuli are species specific has not been examined. Up to now, experiments and comparative scanning electron microscope (SEM) studies on the hairs of the labellum of the flower have not yet yielded sufficient evidence (Pirstinger 1996; Bradshow et al. 2010). It is possible that species-specific tactile stimuli only occur combined with physical contact of chemical substances.

## Pollinator selection on *Ophrys* flowers

### Indirect evidence

The exact agreement between the odorant bouquet of the *Ophrys* flower and the scent of the females of its pollinator species suggests that it has resulted from selection pressure exercised by the males. They bestow those flowers a high reproductive success that best correspond to their expectations of a female partner. As mentioned already, *Ophrys* species with a disjunctive geographical distribution employ the identical pollinator species with identically composed odorant bouquets. (Stökl et al. 2005, 2008, 2009). The same should also apply to optical signals. To prove this, studies have begun which rely on the methods of molecular systematics (Bateman et al. 1997, Aceto et al. 1999). These first results had contributed little to our understanding of *Ophrys* at the species level. The next generation of molecular phylogenetical methods just started with much better results (Breitkopf et al. 2015; Sedeek et al. 2014, 2016; Bateman et al. 2018).

### Direct evidence from experiments on pollination rate

#### The effects of height of flower stem and male-patrolling altitude

**Fig. 3:**
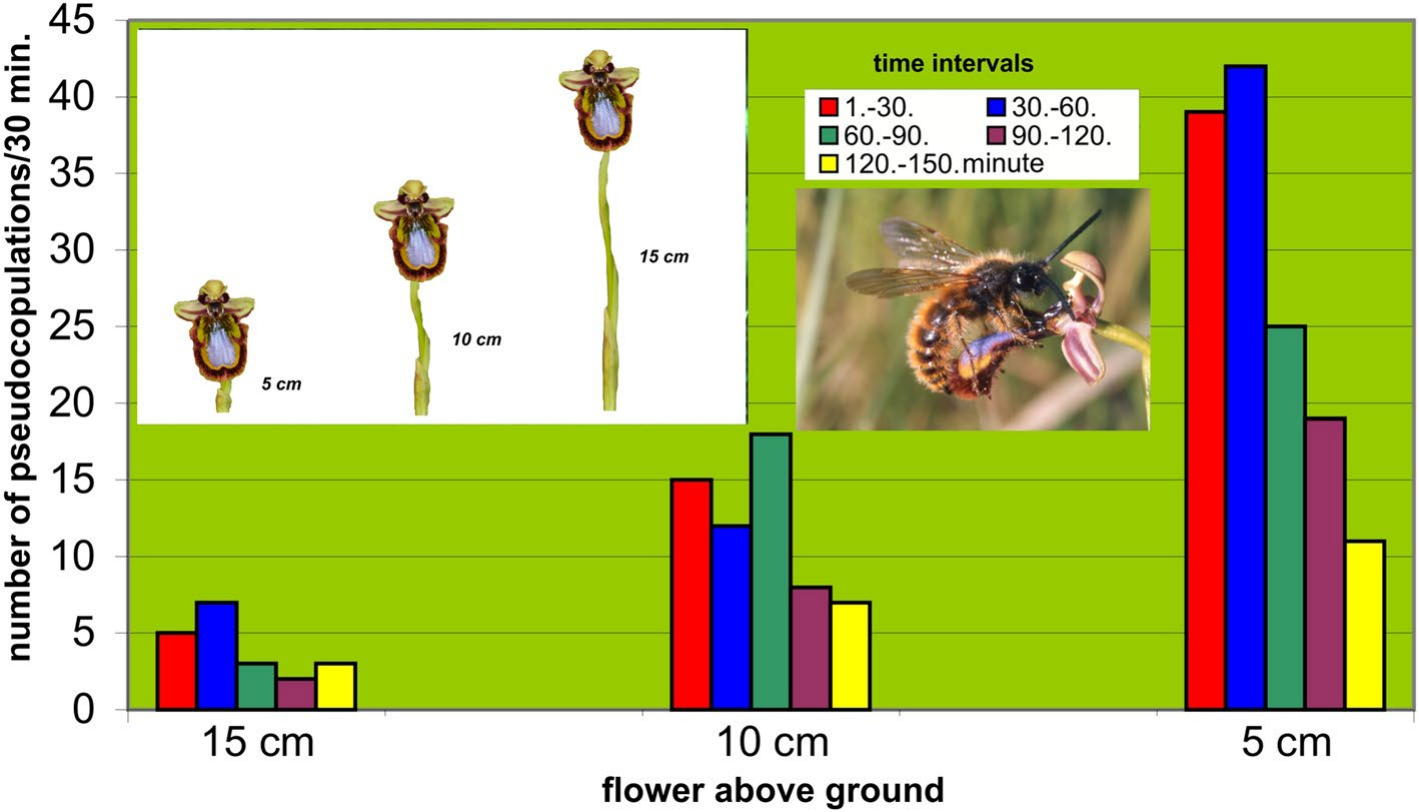
In some *Ophrys* species (here *O. speculum*), the position above ground is under selection. In this experiment, three plants with a flower at different height (5–15 cm) were presented in different swarming areas of males of the pollinator wasp *Dasyscolia ciliata* (Hymenoptera, Scoliidae) and the numbers of pseudocopulations on these flowers counted within 150 min. The flowers near the ground (5 cm) were much preferred (modified from Paulus and Gack 1981, Paulus 2006). Experiments in South Spain near Torremolinos in the years 1976–1981

**Fig. 4:**
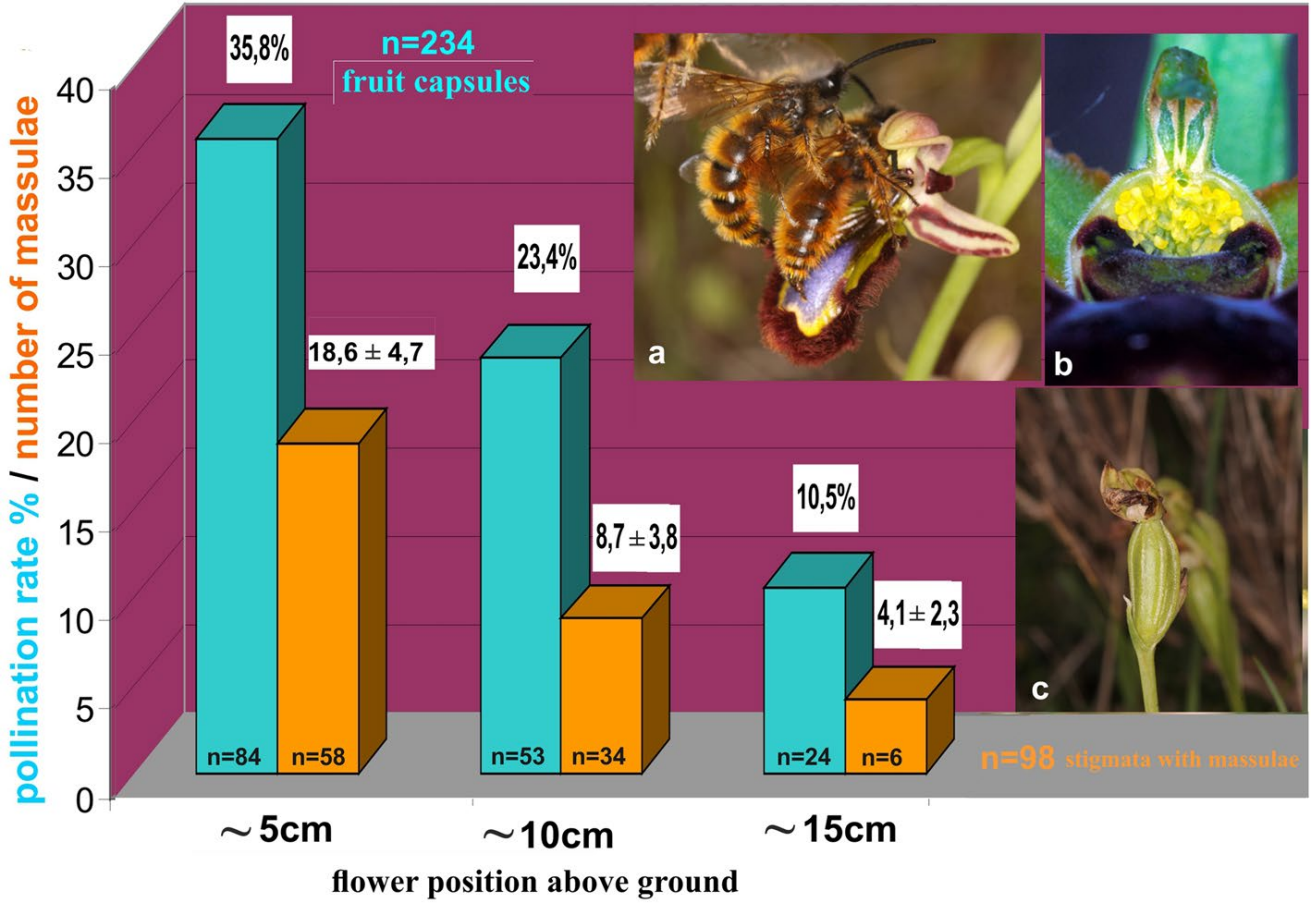
Pollination success in *Ophrys speculum* depending on the position of flowers above ground as indicated by the number of fruiting capsules (blue bars). Observations in Mallorca and Tunesia in 1997–2001 (Paulus and Ettenauer, unpublished) (*n* = 234 flowers of 67 plants). The flowers nearest to the ground had the most pollination success. It means that from 84 flowers in about 5 cm height 35.8% had thickened fruit capsules. This is also reflected by the numbers of massulae attached to the stigmata (orange bars) and deposited by males carrying pollinaria (*n* = 98 flower stigmata). **a***Dasyscolia ciliata* males during pseudocopulation with an *Ophrys speculum* flower (Mallorca 7.4.2009 fot. H.Paulus). **b** Flower stigma pollinated with many massulae by pseudocopulating males. **c** After successful pollination fruit capsules contain about 120,000 seeds (modified from Paulus 2007)

To be sure that pollinators exercise selection on the flowers, males must be shown to affect the reproductive rate of the orchid they choose. This presupposes that the flowers within a certain population are variable and that the males react with different frequencies of approach flights and landings (Paulus 1988, 2007).^1^ Paulus and Gack (1981) were able to show that even the flower height above ground is a relevant factor for *Ophrys speculum*. Pollinator males of the scoliid wasp *Dasyscolia ciliata* were presented flowers simultaneously at heights of 5, 10 and 15 cm above the ground. The numbers of landings with associated pseudocopulation behavior were registered over a period of 150 min in 30-min intervals (Fig. 3). The lower the flower was presented the more frequently it was chosen. This agrees with the observation that males search for females by patrolling just above the ground. In fact, *Ophrys speculum* belongs to those species that are characterized by an inflorescence close to the ground. Direct evidence of reproductive success was found by counting the number of thickened seed capsules in different populations over several years. The undermost flowers had the highest numbers of thickened seed capsules (Fig. 4). The agreement between low plant growth and low levels of the male search flight does not apply to other *Ophrys* species. Their pollinator males generally patrol around bushes and above well-grown fields.

#### How often do males visit Ophrys flowers?

It has been noticed on many occasions that although males are intensely attracted to the flowers, their interest rapidly recedes (Paulus et al. 1983; Paulus 2006, 2007; Streinzer et al. 2009; Rakosy et al. 2012; Stejskal et al. 2016). In a series of learning experiments two hypotheses were tested.

1. After visitation and especially after pollination flowers quickly become unattractive and stop odorant production.
2. The males learn to identify individual flowers and avoid repeated visitations like they do with rejecting true females. They should do this also with an *Ophrys* flower because the probability to get even a successful copulation with a true female is very low and a trial to copulate usually does not imply sperm transfer.

To identify individual flowers by male pollinator bees, two sorts of signals, the individual odorant bouquet and the individual drawing pattern on the labellum, can be learned. Learning experiments gave the answer as to the value of these hypotheses.

**Fig. 5:**
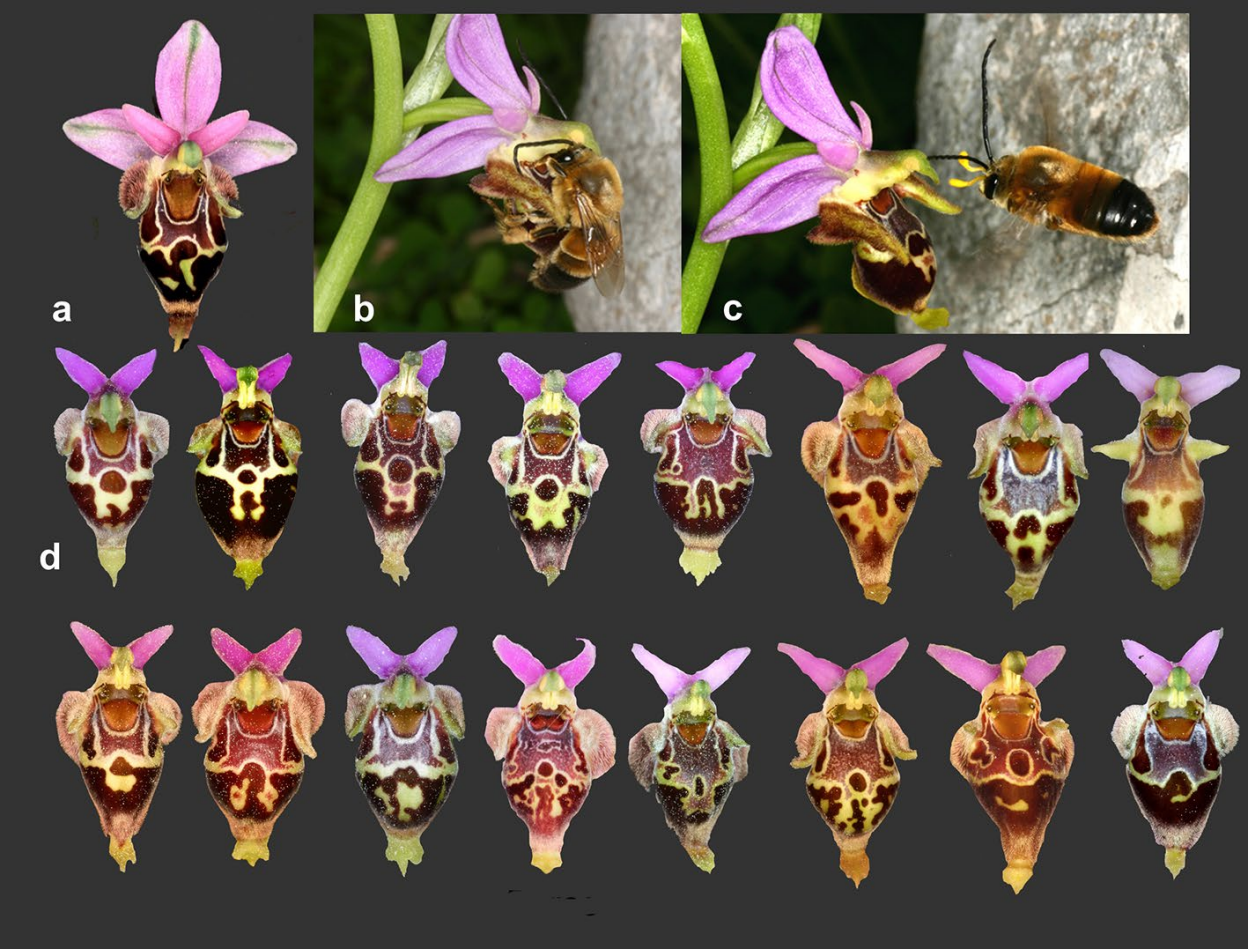
Intraspecific pattern variation of flowers of *Ophrys heldreichii* from Crete (Greece). Each flower lip has a different drawing pattern which also reflects in the UV and can be learnt by pseudocopulating males of *Eucera berlandi*. **a** Flower of *Ophrys heldreichii*, **b** pseudocopulating male of *Eucera berlandi*, **c** male of *Eucera berlandi* having just visited the *Ophrys* flower and removed the pollinaria

Females sometimes reject males which then never try again to copulate with this specific female. For the sweat bee *Lasioglossum* Ayasse et al. (2000) summarized the evidence that males learn to avoid such female partners, recognizing them individually. The males learn the individual odorant bouquet of each female. Learning of individual flowers presupposes that these are individually different as well (Paulus 1988, 2007; Stejskal et al. 2016) (Fig. 5).

Corresponding learning experiments were performed with *Ophrys heldreichii* (Crete), *O. scolopax* (S. Spain) and *O. tenthredinifera* (Tunisia). Each species is pollinated by different *Eucera* species. The procedures were as follows.

**Fig. 6:**
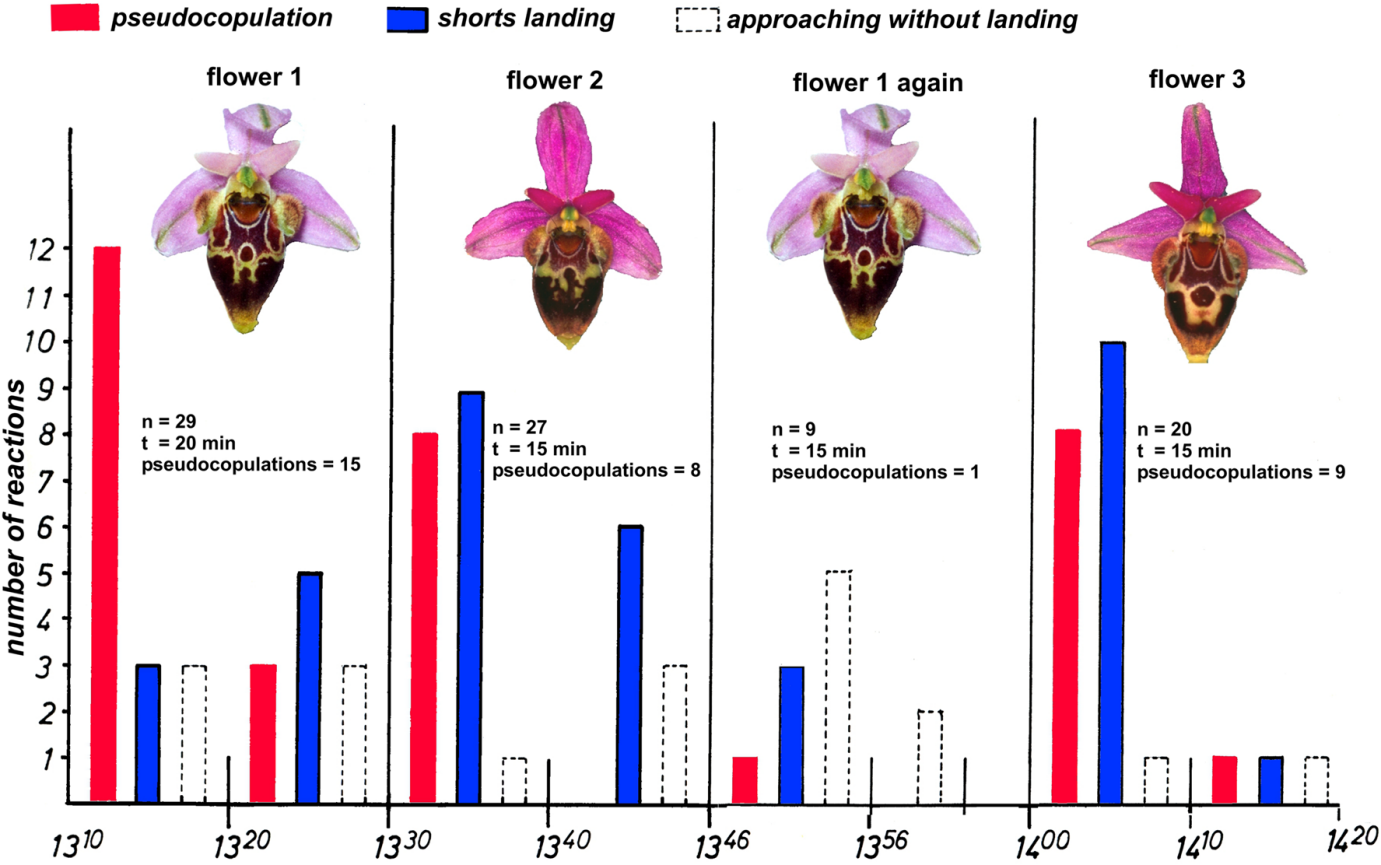
A typical learning experiment with single flowers of *Ophrys heldreichii* from Crete and its pollinator males, the long-horned solitary bee *Eucera berlandi* (Apidae s.str., Eucerini). The flower was presented in a swarm area of female seeking male bees. About 10–15 males are flying within such a territory mixed with some non-territorial males which patrol from territory to territory. The number of the males’ pseudocopulation efforts was counted for 10-min intervals. After 15–20 min, a different flower from another plant was offered. After 10–15 min, the males did not further land or even approach the flower. They recognize each flower individually. Flower 1, which was again presented after 30 min, was visited by one male only. However, the males promptly react again when a new flower, not seen before, is presented

1. One potted plant each of *Ophrys scolopax* (S. Spain), *Ophrys heldreichii* (Greece, Crete) and *Ophrys tenthredinifera* (Tunesia) was placed in the respective male swarm area with about 10–12 males and the number of landings (pseudocopulations and short landings) was measured. The results were very consistent. At first, and very quickly, nearly all of the males in the swarm attempted to copulate with the various flowers of their plant. Within 15–30 min their interest waned so that no further landings were registered. To exclude spatial learning, we repositioned the orchids several times within the swarm areas.
2. In a subsequent experiment, the orchid plant originally presented was replaced with a new specimen of the same species. Immediately, the rate of approaches and landings dramatically increased again and after a short period fell to zero again. This procedure could be repeated two or at most three times before the males completely lost interest.
3. In still another experiment, the plants tested in one area of male pollinator activity were brought to a nearby second swarm area with different males of the same species and naive regarding *Ophrys* flowers. Their behavior was essentially the same as that shown in the previous experiment.
4. When these males were offered a plant that had already been presented to them, the numbers of landings had been in all cases very low (Fig. 6).

From these results, the following conclusions can be drawn:

1. Hypothesis 1 does not apply. The attractiveness of the flowers does not decline simply by the fact that they have been previously visited. Instead, the flowers release immediate copulation attempts when presented to inexperienced males.
2. Possibly, the male bees mark the flowers they visit with a scent (anti-aphrodisiac) (Kukuk 1985). To exclude the possibility that males mark with a species-specific or even an individual odorant, we presented them plants with all flowers sliced lengthwise in half. When the numbers of landing reached zero, all half flowers were exchanged by their respective halves. In all cases, the number of landings did not increase. When males are subsequently offered flower-halves from a different plant individual, these were immediately chosen.
3. Much speaks for hypothesis 2. Obviously male bees remember individual flowers and avoid them later. Accordingly, each and every plant must differ individually and these differences are recognizable to the males (Paulus et al. 1983; Paulus 1988, 2007; Stejskal et al. 2016).

We know that the male bees are able to distinguish flowers of different plant specimens, but in these experiments both types of signals (odorants and visual signals) had been simultaneously presented. In further experiments we tested single flowers.

Both visual and olfactory signals provide a basis for the bees’ memory and learning. The labellum pattern in species of the *Ophrys holosericea*-*oestrifera* group is exceedingly variable and complex—hardly any individual plants have identical patterns (Fig. 5). The flowers of the same inflorescence, however, have virtually identical patterns. To examine male memory, we tested the ability to distinguish between two flowers simultaneously presented to flying male bees. If males are able to distinguish between them, and if we assume it is of value to them, then the landing number should reflect differences between the flowers. A preference by males may be based on vision that is on the lip pattern of the flowers, and/or on the odorant bouquet (Paulus 2006, 2007). The outcome of two types of such experiments was as follows.

1. *Two flowers from the same plant individual* Expectedly, the bees will not distinguish the flowers from each other. The flowers were exchanged left and right every minute to exclude preference for one side or the other. The number of landings on each flower was recorded.

2. *Two flowers from different plant individuals* One can expect that the flowers will be distinguishable from each other and that the number of landings will differ significantly.

**Fig. 7:**
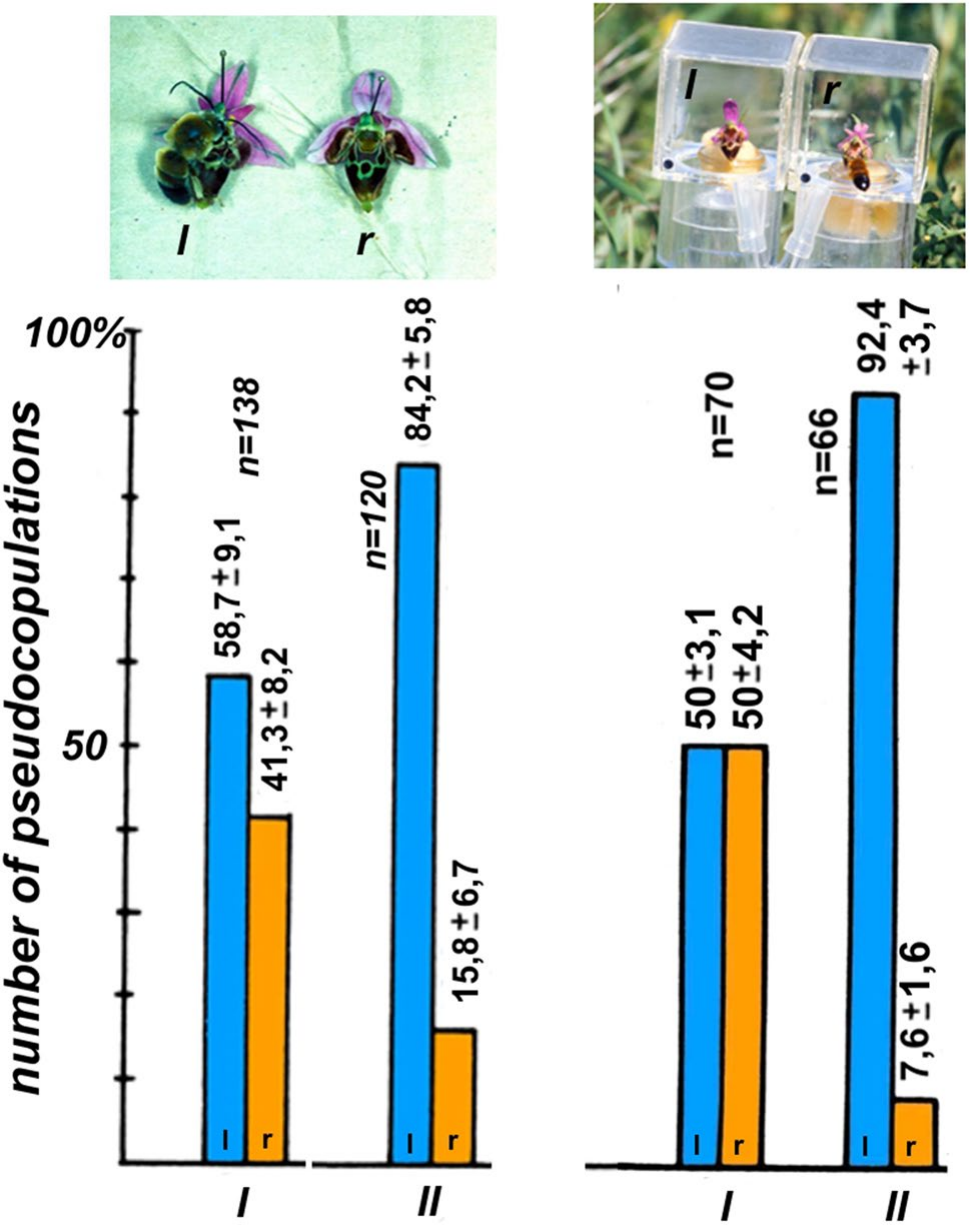
Experiments with two flowers of *Ophrys heldreichii* in Crete (Greece) to test whether males of *Eucera berlandi* are able to distinguish flowers of the same plant (test series I) and flowers of two different plants (test series II). Series I: two flowers presented open with both visual and olfactory signals accessible to the bees. Series II: flowers inside plexiglass boxes with a UV-permeable front side and barring the bees from flower olfactory stimuli. To attract the male bees, smell of *Ophrys heldreichii* flowers cumulated in a box beforehand was blown to the right and left sides of the front glasses. To prevent side preferences (because of the wind) each 10 min. flower position was exchanged. Whereas in test series I male bees could land on the flowers, in test series II strong attempts to land by tipping at the front glass were counted as attempts to pseudocopulate (ps) and short tips as trials to land (sl). Approaches to the flowers without landing were counted as well (ap). Flowers of the same plant individual and presented open are distinguished only weakly and caged flowers, with the visual cues only, not at all. In contrast, flowers of different plants are well distinguished in both situations. Obviously, male bees are able to select between different plant specimens. Cumulation of several test series in different years. Ps, pseudocopulations; sl, short landings; ap, approaching without landings (modified from Paulus 2007)

The actual results of these two experiments are presented in Fig. 7. They confirm the predictions. Males were much less able to distinguish between two flowers from the same inflorescence (ratio of number of landings 1 to 2, than between two flowers from two different plant individuals (ratio 1 to 9).

Remarkably, flowers on the same inflorescence had a landing rate of 1–2 and not 1–1, which means that between these two flowers there was a difference, which possibly came from a difference of odorants. Since the above experiments could not decide whether the males use visual or olfactory cues, another experiment was performed in which the males had to rely only on visual signals. To this end, the flowers were placed in clear transparent plastic boxes, the sides of which were permeable to ultraviolet light. In this manner the scent of the flower could not disperse. Since males generally do not respond to optical signals of *Ophrys* alone, flower scent was pumped to the outside of the boxes with a Y-shaped tube. Thus, each box was surrounded by the same scent and male choice could be based only on optical stimulation. Since the males were prevented from actually landing on the flower, we counted as a positive reaction what appeared to be an obvious attempt to land.

The two situations tested were the following. 1. *Two flowers from the same plant individual* The expectation is that they will not be distinguished from each other since they are visually identical. 2. *Two flowers from two different plant individuals* We should expect here roughly the same difference as in the open flower experiment. The results of these tests were similar to those obtained before. The two flowers from different plants were clearly chosen at different rates. The two flowers from the same plant individual were not distinguished at all. Two conclusions can be drawn from these results. (i) Plants of the same species (in our case, *Ophrys heldreichii*) exhibit individual variation regarding their scent. Thus, in addition to a highly variable visual pattern on the labellum, there is an individuality of scent regarding individual plants of the same species. (ii) Individuality of scent is even apparent on different flowers of an inflorescence. Analyses on the chemical components of the scent of *Ophrys sphegodes* verify the conclusions reached by field experiments. The studies by Ayasse et al. (1997, 2000), Schiestl et al. (1997a) b, (2000) and, in particular, Schiestl and Ayasse (2001) demonstrated not only the existence of a species-specific scent, but in addition to an individual scent for individual plants even one for the individual flowers within the same inflorescence. The results were confirmed in various biotests performed on the males of *Andrena nigroaenea*. The difference of the rate 1–2 in the experiments with open flowers could imply that there is a small age-dependent odorant variability.

### Optimal pollination and avoidance of self-fertilization

The *Ophrys* type of individual variation in olfactory and visual signals can best be explained in relation to an optimization of the rate of pollination, since differences in landing numbers affect the orchid’s reproductive success. It must also be recognized that, in general, orchids are prone to greater problems with self-pollination than other flowering plants. The invention of pollinaria was necessary to ensure that with only few visitations of large amounts of pollen can be transferred to the stigma. In this manner, each flower produces an immense amount of seeds. The number of seeds in a single orchid flower ranges between 15,000 and one million (Paulus 1988; Nazarov and Gerlach 1997, and many own countings). Self-fertilization in this kind of pollination system may entail fatal consequences, as self-pollination results in only very few seeds (Detto 1905) and, therefore, large amounts of pollen may be lost. Further special adaptations are necessary to avoid self-pollination. One of these adaptations is the development of deceptive methods which, although they promote the attraction of a potential pollinator, they also discourage repeated visitations from the same pollinator individual by providing no rewards or even by “frustrating” the visitor. This is the “pollinium hypothesis” or “self-pollination avoidance hypothesis” proposed by Paulus and Gack (1981), Paulus and Gack 1990), Paulus (1988, 2006), and reviewed by Nilsson (1992). Since the pollinator males possess learning capacities, they avoid these flowers in the future. The pollinium hypothesis adequately explains the fact that especially Orchidaceae have evolved a wide variety and amount of pollination systems based on deception. Pollination by deception is found in over 75% of all orchid species in the European flora (Paulus 2007) and this might be true for the orchids worldwide. The only other plant group which also has developed pollinia also is the Asclepiadiaceae (milk weeds). Presumably for the same reasons, they have also developed a large number of deceptive flowers (Ollerton and Liede 1997).

To maximize pollination, *Ophrys* flowers have developed additional tricks. As already indicated by choice experiments with pairs of *Ophrys* flowers (Paulus 1988, 2007; Paulus and Gack 1995), the odorant bouquets are not identical. Gas chromatographic analyses show that many *Ophrys* species operate with more or less the same compounds but differ in ratios of alkanes and alkenes in the lip of the flower (Schiestl et al. 1997b; Ayasse et al. 2000). Because of the avoidance reaction of males that have been deceived before, these will fly with increased probability to the next flower of the same inflorescence. In this manner, a second pollinium can be removed and if the bee is already carrying a ripened pollinium from a previous visitation, it will pollinate the second flower. The previously removed pollinia are capable of pollination only after 2–3 min, after the stalk has dried and bends forward.

*Ophrys* flowers have developed clever mechanisms which cause males to regard those flowers as unattractive that have already been pollinated. Since a single pollination event will transfer more pollen than is necessary for the number of ovules (Paulus 1988, 2007), an *Ophrys* flower obtains no benefit from additional visitations or pollination attempts. Anything more than a single pollination event means wasted pollen. The male’s initial interest in the flower should be redirected to other flowers of the same inflorescence which have not yet been visited. This can be achieved in at least two conceivable manners. Since after mating, the female bees halt production of the sexual attractant or even begin to produce an anti-aphrodisiac (Schiestl and Ayasse 2000), the *Ophrys* flowers, too, may respond in a similar fashion.

## Conclusions

There are two methods for proving effects of selection. An *indirect approach* is a cross-lineage approach to understand the phylogenetic history of individuals or higher taxa and the mechanisms that drive it. The phylogenies are important for comparative analyses and usually represented by a phylogenetic tree to distinguish features with single origins (homology, monophyly) from those with multiple origins (homoplasy). According to the phylogenetical analyses of many of the pollination modes, nearly all of them originate in multiple evolutionary ways. Different pollination methods found in closely related species can be explained as a change of selection pressures going along with a change of the pollinators. The most frequent pollinators exert the strongest selection because they generate the highest seed numbers. As selection applies to the difference of reproductive success between two individuals of a species caused by differences in their fitness, these new pollinators will convert the characteristics of the flowers fit for a new pollination mode. Many cases of such evolutionary switches (e.g. from bat to bird pollination or from wind to secondary insect pollination) could be demonstrated.

To demonstrate a more *direct method* to understand selection pressures, the measurement of differences in the reproductive success between individuals of a given *Ophrys* species are chosen as an example. In these sexually deceptive orchids, differences in the frequency of pollinator visits can be examined, which is correlated with differences of the plant’s reproductive success. Condition for studying the efficacy is a high genetic variability related to flower signal variability whose stimulation capacity can be tested. After having been visited by the pollinator for the first time, the flower signals in sexual deceptive orchids were shown to repel the pollinating males to avoid self-pollination. This generated an astonishing learning behavior in pollinating males to avoid just visited flowers. As these males visit only new flowers with different individual signals, they support cross-pollination.

Members of highly specialized pollination modes such as sexual deception are quite different from those with more generalized attraction methods offering rewards.

In all cases, competition for pollinators might be the main selection effort but I suppose that in specialized systems natural and sexual selection are the main factors whereas in communities with many reward offering flower species and many different flower visiting insect species a kind of multilevel selection (Wilson 2010) is more important. Even facilitation might be a better explanation of flower evolution as some recent investigations with community modelling could demonstrate (Kantsa et al. 2017, 2018).

## Footnotes

1 All experiments are observations in the field. Plants have been presented in flying areas of pollinator males and the numbers of reactions counted. The absolute numbers of countings are depending on how many males are flying in a special field. As all reactions by those males are “all or nothing“ reactions and the experimental conditions are variable, I only present the basic numbers of reactions waiving of statistics because there are no differences in the validities.
